# Etiological Spectrum of Bone Marrow Biopsy in Patients With Pancytopenia

**DOI:** 10.7759/cureus.73138

**Published:** 2024-11-06

**Authors:** Inayat Ullah, Dilaram Khan, Vaneeza Qureshi

**Affiliations:** 1 General Medicine, Lady Reading Hospital Peshawar, Peshawar, PAK; 2 Gastroenterology, Lady Reading Hospital Peshawar, Peshawar, PAK; 3 Medicine, Lady Reading Hospital Peshawar, Peshawar, PAK

**Keywords:** aplastic anemia, biopsy, bone marrow biopsy, bone marrow failure, megaloblastic anemia, pancytopenia, thrombocytopenia, thrombocytopenia pancytopenia

## Abstract

Background

Pancytopenia is defined as a decrease in all three hematologic cell lines. The condition is not a disease in itself but a common pathway caused by various etiologies that can be infectious, autoimmune, genetic, nutritional, and/or malignant. Determining the cause of pancytopenia is a challenge and is key in determining the proper treatment regimen and estimating prognosis.

Objective

The aim of this study was to evaluate the etiological spectrum of pancytopenia in patients who underwent bone marrow biopsy at a tertiary care hospital.

Methodology

This study was conducted at the Internal Medicine and Gastroenterology Departments of Lady Reading Hospital, Peshawar, Pakistan, from January 1, 2023, to December 31, 2023. A total of 120 patients with pancytopenia aged 12 to 60 years who underwent bone marrow biopsy were enrolled. Data on age, gender, clinical presentations, and bone marrow biopsy findings were observed.

Results

The mean age was 34.97 years. Males were more frequently affected, 74 (61.7%), than females, 46 (38.3%). Pallor, 86 (71.7%), and weakness, 66 (55.0%), were the most frequent presentations of pancytopenia. The etiological spectrum showed that megaloblastic anemia was present in 48 (40%) of the cases, followed by infection-related changes in 20 (16.7%) and aplastic anemia in 20 (16.7%).

Conclusion

Megaloblastic anemia emerged as the leading cause of pancytopenia in our study, followed by aplastic anemia and infection-related changes.

## Introduction

Pancytopenia is a hematologic disorder caused by a reduction in hemoglobin, platelets, and white blood cells [1,2]. Nevertheless, these criteria mostly rely on factors such as age, gender, ethnicity, and different clinical situations. Leukopenia is mainly observed as neutropenia, while neutrophils make up the majority of white blood cells. Pancytopenia is not considered a standalone disease but rather a symptom that arises from various underlying medical disorders [3]. It is frequently linked to several non-cancerous and cancerous illnesses. Pancytopenia may occur due to reduced cellular production or heightened cellular death. A comprehensive assessment is necessary to determine the root cause of pancytopenia in any individual [4].

Pancytopenia can occur due to various processes, including a decrease in the production of hematopoietic cells caused by the destruction of the bone marrow by toxins or the suppression of normal growth and differentiation of the marrow [5-7].

The composition of the marrow varies depending on the reason. In instances of pancytopenia resulting from primary production abnormalities, the bone marrow typically exhibits a lower-than-normal cellularity. Cytopenias, which occur due to increased peripheral use or death of cells, inadequate hematopoiesis, and bone marrow invasive processes, are typically linked to a normocellular and hypercellular marrow [8,9]. The symptoms that are typically observed are caused by either thrombocytopenia or anemia [9]. Leukopenia is rare when the patient is first diagnosed, but it may turn into the most life-threatening complication as the illness progresses. Typical clinical symptoms include pallor, tiredness, enlarged spleen, swollen lymph nodes, fever, hemorrhage, weight loss, enlarged liver, and yellowing of the skin [10,11].

Performing a bone marrow biopsy serves as an essential diagnostic method for determining the cause of pancytopenia. However, the range of possible causes might vary significantly. Comprehending this range of possibilities is crucial for precise identification of medical conditions, prompt intervention, and enhancing the results for patients. The main goal of this study was to determine the etiological spectrum of bone marrow biopsy in patients presenting with pancytopenia to our hospital.

## Materials and methods

Study design

This descriptive study was conducted at the departments of Internal Medicine and Gastroenterology of Lady Reading Hospital, Peshawar, Pakistan, over a one-year period (January 1, 2023, to December 31, 2023).

Inclusion criteria

We selected 120 patients aged between 12 and 60 years diagnosed with pancytopenia, defined as hemoglobin <10 g/dL, an absolute neutrophil count of <1.5 × 10^9^/L, and a platelet count of <100 × 10^9^/L.

Exclusion criteria

Patients who had received chemotherapy or radiotherapy within three months before the bone marrow biopsy or if their bone marrow aspirate samples were inadequate for diagnosis were excluded from the study.

Sample size

The sample size was 120, which was calculated using the Openepi calculator, taking previous frequency of acute leukemia as one of the etiologies of pancytopenia (8%) [12], margin of error 4.8%, and confidence interval 95%.

Ethical consideration

The Institutional Review Board of the Lady Reading Hospital approved the study (approval reference number: 280/LRH/MTI dated 15/12/2022) and ensured that it complied with all applicable ethical standards. Patient information anonymity was ensured, and all study procedures followed the guidelines outlined in the Declaration of Helsinki.

Data collection procedure

Patients were enrolled in the institute’s indoor and outdoor internal medicine and gastroenterology sections. Demographics (thorough medical history), physical examination, biochemical indices, and imaging were documented.

All the patients underwent bone marrow biopsy for further evaluation of pancytopenia. Bone marrow biopsy was performed using standard techniques. The samples were processed and analyzed by experienced hematopathologists. Special stains and immunohistochemical markers were used as needed to reach a definitive diagnosis.

Data analysis

SPSS Version 24 (IBM Corp., Armonk, NY) was used for all statistical analyses.

## Results

A total of 120 patients, 74 (61.7%) males and 46 (38.3%) females, were enrolled in this study, with a male-to-female ratio of 1.60:1. The age of the patients ranged from 12 to 60 years, with a mean age of 34.97±12.709 years (Table 1).

**Table 1:** Distribution of patients according to age (n=120)

Age (years)	Number	Percentage
12-20	18	15
21-30	34	28.3
31-40	28	23.3
41-50	26	21.7
51-60	14	11.7

We examined various clinical presentations of pancytopenia. Pallor was the most frequent presentation noted in 86 (71.7%) of the cases. Weakness/aches and pains were present in 66 (55.0%) patients. Fever was observed in 34 (28.3%) patients. Splenomegaly was present in 22 (18.3%) patients, hepatomegaly was seen in 14 (11.7%) patients, and bleeding tendencies were noted in 18 (15%) patients (Table 2).

**Table 2:** Clinical presentations of pancytopenia

Variable	Number		Percentage (%)
Pallor	Yes	86	71.7
	No	34	28.3
Weakness/body aches	Yes	66	55
	No	54	45
Fever	Yes	34	28.3
	No	86	71.7
Bleeding tendency	Yes	18	15
	No	102	85
Splenomegaly	Yes	22	18.3
	No	98	81.7
Hepatomegaly	Yes	14	11.7
	No	106	88.3

The etiological analysis of pancytopenia showed that megaloblastic anemia was present in 48 (40%) patients, followed by infection-related changes in 20 (16.7%) and aplastic anemia in 20 (16.7%). Acute leukemia was present in eight (6.7%) patients, while chronic leukemia, hypersplenism, and myelodysplastic syndrome were found in 4 (3.3%), 4 (3.3%), and 14 (11.7%), respectively (Table 3).

**Table 3:** The etiological spectrum of bone marrow biopsy

Variable	Number	Percentage (%)
Acute leukemia	8	6.7
Aplastic anemia	20	16.7
Chronic leukemia	4	3.3
Hypersplenism	4	3.3
Infection-related changes	20	16.7
Megaloblastic anemia	48	40
Myelodysplastic syndrome	14	11.7
Others	2	1.7
Total	120	100

## Discussion

In the present study, which included 120 patients, the age distribution ranged from 12 to 60 years, with a mean age of 34.97 years. The majority of patients were in the age group of 21-30 years (28.3%), indicating that pancytopenia is more prevalent in this age group. A study conducted by Gajbhiye et al. showed that the majority of their patients were in the age group of 25 to 30 years [12].

The gender distribution in the current study shows a male predominance, with 61.7% of patients being male and 38.3% being female. This is consistent with findings of Zulfiqar et al., where they found that male patients had higher frequency than female patients. This shows that males appear to be more frequently affected by pancytopenia than females [13].

In terms of clinical presentation, pallor showed higher frequency, observed in 71.7% of patients. This was followed by weakness or body aches in 55% of patients and fever in 28.3%. Splenomegaly and hepatomegaly were found in 18.3% and 11.7% of patients. Similar findings were reported by Memon et al. in their study, where pallor was the most frequent clinical feature in 87% of patients [14]. However, Zulfiqar et al. showed that fever was more commonly reported, affecting 54.8% of patients [13]. Gajbhiye et al. reported that fatigue was the common feature of pancytopenia [12].

The relatively lower incidence of fever in the current study compared to Zulfiqar et al.’s study could be due to the different etiological factors prevalent in the study populations. Pallor and weakness were common signs across most studies [15,16].

The etiological spectrum in our study identified megaloblastic anemia as the most frequent etiology of pancytopenia, accounting for 40% of cases. This finding is consistent with other studies, including Zulfiqar et al., who reported megaloblastic anemia in 29.6% of their patients [13]. Another study conducted in Pakistan by Farooque et al. also reported that megaloblastic anemia was the leading etiology of pancytopenia in their series [17]. The high prevalence of megaloblastic anemia underscores the impact of nutritional deficiencies, particularly in developing regions where dietary inadequacies are more common.

Other significant causes identified in the current study included infection-related changes (16.7%) and aplastic anemia (16.7%). Myelodysplastic syndrome (11.7%) and acute leukemia (6.7%) were also notable etiologies. These results are in agreement with the broader spectrum of causes observed in Zulfiqar et al.’s study, where infection-related changes were the second most common cause (26.6%), followed by aplasia (10.3%) [13]. Contrary to our results, Memon et al. in their study showed a higher incidence of aplastic anemia (23.9%) as the leading cause, with megaloblastic anemia accounting for 13.04% [14].

Limitations

This was a single-center study and may not the true representative of the whole community. Therefore, large-scale multi-center studies are needed to know the exact etiological spectrum of bone marrow biopsy in patients with pancytopenia.

## Conclusions

In conclusion, our study showed that megaloblastic anemia is a leading, non-malignant cause of pancytopenia, followed by aplastic anemia and infection-related changes. It was through the bone marrow aspiration biopsy examination that such a wide spectrum of diagnoses was made in patients presenting with the single finding of pancytopenia. Hence, bone marrow aspiration biopsy is useful diagnostic tools in evaluating pancytopenia. Effective management of pancytopenia requires early diagnosis of these etiologies for timely treatment, ultimately improving patient outcomes.
